# Reducing morbidity with surgical adhesives following inguinal lymph node dissections for the treatment of malignant skin tumors

**DOI:** 10.3205/iprs000084

**Published:** 2016-01-20

**Authors:** Peter. L. Stollwerck, Dominik Schlarb, Nicole Münstermann, Sebastian Stenske, Christoph Kruess, Gerhard Brodner, Björn Dirk Krapohl, Albrecht F. Krause-Bergmann

**Affiliations:** 1 Department of Plastic and Aesthetic Surgery, Hand Surgery, Fachklinik Hornheide, Münster, Germany; 2 Department of Anesthesiology, Intensive Care and Pain Medicine, Fachklinik Hornheide, Münster, Germany; 3 Department for Plastic Surgery and Hand Surgery, St. Marien Hospital, Berlin, Germany; 4 Center for Musculoskeletal Surgery, Charité – University Medicine Berlin, Germany

**Keywords:** skin tumor, surgical adhesive, complications, lymph node dissection

## Abstract

**Background:** Inguinal lymph node dissection (ILND) is associated with a high rate of morbidity. To evaluate the clinical benefit of surgical adhesives to reduce complications in patients undergoing ILND, we compared the use of TissuGlu^®^ Surgical Adhesive and ARTISS^®^ fibrin sealant with a control population.

**Material and methods:** We conducted a retrospective analysis of patients undergoing ILND for metastatic malignant skin tumors at one hospital, Fachklinik Hornheide (Münster, Germany), from January 2011 through September 2013, assessing 137 patients with a total of 142 procedures.

**Results:** Complications occurred in 22/60 procedures in the TissuGlu group (TG), in 8/17 in the ARTISS group (AG), and in 29/65 in the control group (CG). Prolonged drainage and seroma were recorded in 16 (26.7%), four (23.5%), and 26 (40%) respectively (non-significant). TG showed less extended drainage vs. CG (p=0.082). Mean daily drain volumes were significantly lower in AG vs. CG (p=0.000). With regard to wound infection, there was a 15% reduction in TG and 74% increase in AG group. Revision surgery was reduced by 36% in TG and increased by 54% in AG. Mean daily drain volumes were significantly lower in AG vs. CG (p=0.000). Mean total post-operative drain volume was lower in TG and AG vs. CG (p<0.001 among groups, CG vs. TG p<0.001, CG vs. AG p<0.001). The mean body mass index (BMI) was significantly higher in patients with complications, 29.4±5.8 vs. 25.3±4.1 (p=0.000).

**Conclusion:** The use of TissuGlu in our ILND patients was associated with a reduction in post-operative wound related complications and the need for revision surgeries compared to the control group. Daily drainage was significantly lower within the first 7 post-operative days with the use of ARTISS, but the benefit was lost due to the higher occurrence of wound infection and revision surgery. BMI above 29 is a risk factor for complications following ILND.

(Level of evidence: level IV, retrospective case study)

## Zusammenfassung

**Hintergrund:** Inguinale Lymphknotendissektion geht mit hoher Morbidität einher. Um den klinischen Nutzen von Gewebeklebern zu bewerten, verglichen wir die Verwendung von TissuGlu^®^ und ARTISS^®^ mit einer Kontrollgruppe.

**Material und Methoden:** Wir führten eine retrospektive Analyse von Patienten, die sich einer inguinalen Lymphknotendissektion im Rahmen eines metastasierenden malignen Hauttumorleidens an einem Krankenhaus, der Fachklinik Hornheide (Münster, Deutschland), unterzogen. Im Zeitraum Januar 2001 bis September 2013 schlossen wir 137 Patienten mit insgesamt 142 Eingriffen ein.

**Ergebnisse:** Komplikationen traten in 22/60 Eingriffen in der TissuGlu-Gruppe (TG), in 8/17 in ARTISS-Gruppe (AG) und in 29/65 in der Kontrollgruppe (CG) auf. Verlängerte Drainage und Serome lagen in 16 (26,7%), vier (23,5%) bzw. 26 (40%) vor (non-signifikant). TG führte zu einer längeren Drainagedauer Vergleich zu CG (p=0,082). Das durchschnittliche tägliche Drainagevolumen war signifikant niedriger in der AG- vs. CG-Gruppe (p=0,000). Im Hinblick auf Wundinfektionen waren eine 15%-Reduktion in der TG-Gruppe und eine 74%-Erhöhung in der AG-Gruppe zu verzeichnen. Die Notwendigkeit zur chirurgischen Revision wurde um 36% in der TG-Gruppe gesenkt und um 54% in der AG-Gruppe. Das mittlere tägliche Drainagevolumen war signifikant niedriger in der AG- vs. CG-Gruppe (p=0,000). Das mittlere postoperative Gesamtdrainagevolumen war niedriger in der TG- und AG- vs. CG-Gruppe (p<0,001 im Vergleich zwischen den Gruppen TG vs. AG bei den Gruppen CG vs. TG p<0,001 und bei CG vs. AG p<0,001). Der Body Mass Index (BMI) lag im Mittel signifikant höher bei den Patienten mit Komplikationen, 29,4±5,8 vs. 25,3±4,1 (p=0,000).

**Schlussfolgerung:** Die Verwendung von TissuGlu war bei unseren Patienten mit inguinaler Lymphknotendissektion im Vergleich zur Kontrollgruppe mit einer Reduktion postoperativer Wundheilungsstörungen und mit einer Verminderung der Zahl chirurgischer Revisionen assoziiert. Das tägliche Drainagevolumen in den ersten sieben postoperativen Tagen war signifikant geringer nach Verwendung von ARTISS; dieser Vorteil wurde jedoch aufgewogen durch das vermehrte Auftreten von Wundinfektionen und Revisionseingriffen. Ein BMI über 29 stellte einen Risikofaktor für Komplikationen nach inguinalen Lymphknotendissektion dar.

(Evidenzlevel: Level IV, retrospektive Fallstudie)

## Introduction

Inguinal lymph node dissection (ILND) for malignant melanoma is the gold standard in the current guidelines for the treatment of node-positive melanoma [1], [2], [3]. Unfortunately, this procedure is frequently associated with development of significant post-operative complications such as seroma formation, infections, and slow recovery, negatively impacting patient short-term quality of life and also increasing hospital costs [4], [5], [6], [7], [8], [9], [10].

A recent prospective study of melanoma patients undergoing ILND reported that 77% of patients developed post-operative complications [7]. This included 55% of patients developing infections, 53% having wound dehiscence and 28% experiencing seroma formation. Seroma formation was associated with infectious complications in one third of these cases. Obesity (BMI >30 kg/m^2^) was associated with an 11-fold increase in the risk of wound complications. 

The complications in our study population were defined as prolonged drainage/seroma, wound infection and the need for revision surgery. In our efforts to further reduce post-operative complications following ILND, we began exploring the use of a novel tissue adhesive (TissuGlu^®^ Surgical Adhesive, Cohera Medical, Inc., Pittsburgh, Pennsylvania, USA). TissuGlu is a synthetic, high strength lysine-derived urethane adhesive for internal use, designed to facilitate natural healing of tissues following surgical procedures involving extensive undermining of tissue and the creation of tissue flaps as in abdominoplasties [11]. The adhesive acts to bond tissue layers together thereby reducing dead space. Upon exposure to moisture in the biological environment, this biocompatible adhesive bonds to both, to tissue and to itself to form a fully cured polymeric network. During the further healing process, hydrolysis leads to breakdown of the polymer and absorption by the body. For the same reasons, we also used ARTISS^®^, human derived two component fibrin sealant (Baxter Germany GmbH, Unterschleißheim). This two component human fibrin is applied by a spray applicator and is indicated to adhere autologous skin grafts to surgically prepared wound beds or to adhere tissue flaps during facial rhytidectomy surgery (face-lift). 

This report reviews the results of our clinical use of TissuGlu and ARTISS to date in our ILND patients, with a focus on post-operative complication rates, and compares these results to other patients undergoing this standard procedure without the use of adhesives at our center. 

## Material and methods

This study was approved by the local ethics committee. We conducted a retrospective analysis of post-surgical outcomes for patients who underwent inguinal lymph node dissection for metastatic malignant skin tumors at our hospital. We included a total of 137 consecutive patients who all underwent an ILND procedure using the longitudinal incision and with ligation of the long saphenous vein between January 2011 and September 2013. Three groups were defined as TissuGlu group, ARTISS group and standard care group (further called control group). The control group, n=63 was treated between 2011 and 2012. The second group underwent the same procedure with the addition of a lysine-based urethane adhesive or a fibrin sealant during 2012 and 2013 (TissuGlu group, n=57, ARTISS group, n=17). Two patients in the control group were excluded as a result of incomplete data. 

Surgical and post-operative management was identical for all patients with the exception of the use of TissuGlu or ARTISS fibrin sealant. Our standard procedure for these patients included elastic wrapping of the affected leg, including the groin and hip region followed by a three day bed rest. According to our standard procedure, suction drains were removed when the daily drainage volume per drain was <30 ml in 24 hours. Otherwise they were to be removed by day eight regardless of drainage volume and replaced by a passive drain (Easy Flow silicone drain) for patients who experienced persistent drainage. In these cases patients were sent home with the passive drains in place and daily drain volume and date of drain removal were not available. Ongoing drainage at day 8 and beyond was considered a complication for the purposes of this study since it necessitated additional clinical intervention with the minimum being later removal of the passive drains.

**Figure 1:**
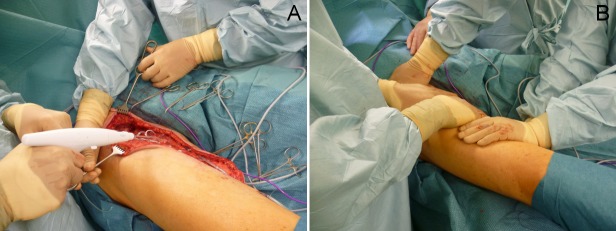
A) Application of TissuGlu adhesive on the fascia lata. B) Pressing of skin flaps against the fascia.

For the TissuGlu patients, following lymph node dissection the adhesive was applied to the fascia lata in the area to be covered by the skin and subcutaneous tissue. The skin was then carefully pulled over the top of the fascia to avoid smearing the glue and placed into its final position. The skin was then pressed against the underlying fascia and closed with subcutaneous sutures and skin staples (Figure 1 (Fig. 1)). For ARTISS patients 2 ml of the two-component human fibrin sealant, containing human thrombin and fibrinogen was applied to the fascia lata with a spray applicator. In these cases, the skin was pressed against the fascia as well and wound closure performed in the identical manner. Surgery was performed by four consultant plastic surgeons of our department. 

Data for the analysis was obtained following a retrospective chart review which included patient demographics (age, gender, height, weight, BMI, and date of operation) and post-surgical outcomes including the number of post-operative hospital days, daily drain volume, days until suction drain removal, need for extended drainage (at day 8 and beyond) and post-operative complications (infection, prolonged drainage, wound revision). 

Statistical analysis was performed using IBM^®^ SPSS^®^ version 22. P-values ≤0.05 were considered as statistically significant. Nominal scale variables were described using relative and absolute frequencies, and the χ^2^ test was used to assess differences between groups. Fisher’s exact test was used if matched cells were rare (expected frequencies less than five). Variables with interval or rational scales were described as means and standard deviation. One-way analysis of variance with post hoc Scheffé test or repeated-measures analysis of variance was used to compare groups. Interval scaled variables were described as mean and standard deviation or as absolute and relative frequencies. To give a clinical impression of the effect of TissuGlu and ARTISS in the prevention of complications such as revision surgery, infection, and extended drainage, we calculated the number needed to treat (NNT). 

## Results

Complete data were available for a total of 137 patients. This included 57 patients in the TissuGlu group, 17 patients in the ARTISS group and 63 patients in the control group. Three patients in the TissuGlu group and two patients in the control group underwent bilateral procedures. Total numbers of procedures were therefore 60, 17, and 65 respectively for the three groups (total: 142). 127 patients (89%) were treated for cutaneous malignant melanoma, 6 (4%) for squamous cell carcinoma, 6 (4%) for Merkel cell carcinoma, 1 for Bowen carcinoma (1%), 1 for leiomyosarkoma (1%), and 1 for B-cell type leukemia. 132 of the cases (93%) underwent sentinel node biopsy before ILND. Patient data were observed over a mean post-operative period of 17±10 months (range 1 to 33 months). Average hospital stay of all patients was 9.5±5.2 days (range 3 to 30 days). There were no statistical differences in the demographics and co-morbidities between the three groups (Table 1 (Tab. 1)).

### Complications

**Figure 2:**
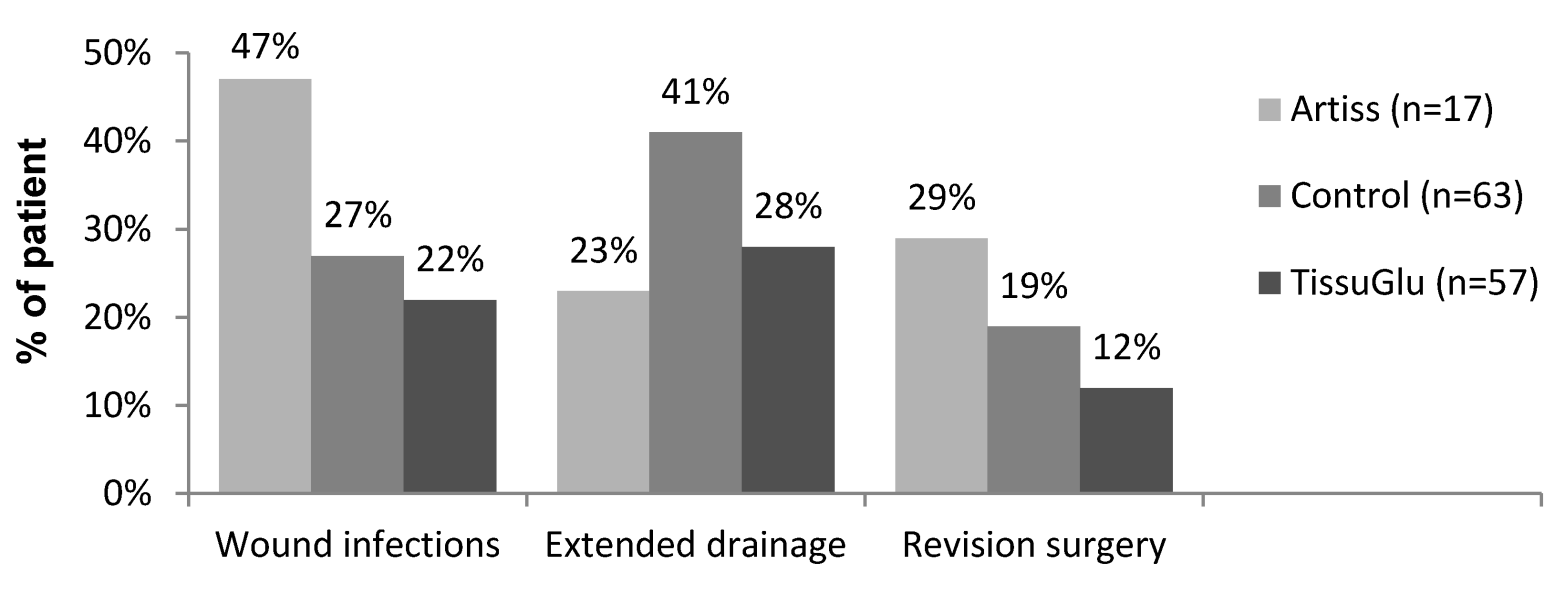
Post-operative wound-related complications

In 59 of all 142 ILND procedures (41.5%) at least one or more complications occurred in the course of post-operative period. These were extended drainage, wound infections, and the need for hospital re-admission with revision surgery. The results are summarized in Figure 2 (Fig. 2). Overall post-operative complications occurred following 22/60 (36.7%) of the procedures in the TissuGlu group, 8/17 (47.1%) in the ARTISS group compared to 29/65 (44.6%) of procedures in the control group (non-significant). This represents a 16% reduction in the TissuGlu treatment group and an increase of 2% in the ARTISS group as compared to the control group.

### Extended drainage

Extended drainage was noted in 16/60 (26.7%) of the procedures for the TissuGlu group, 4/17 (23.5%) in the ARTISS group, and 26/65 (40%) of procedures for the control group (non-significant). This leads to a 32% reduction in the need for extended drainage in the TissuGlu group and 43% reduction in the ARTISS group. The difference between the TissuGlu and control group showed a trend (p=0.082) towards less extended drainage in the TissuGlu group.

Seventeen of 63 patients (26.2%) in the control group experienced continued drainage requiring the use of a post-discharge passive drain compared to 12/57 patients (20%) in the TissuGlu group and 3/17 (17.6%) in the ARTISS group (non-significant).

### Wound infection

Thirteen of 57 patients (22%) in the TissuGlu group, 8/17 (47%) in the ARTISS group, and 17/63 (27%) in the control group developed post-operative wound infections which needed to be treated with antibiotics (ns). This meant a 15% reduction in post-operative infections in the TissuGlu group and 74% increase in the ARTISS group.

### Revision surgery

**Figure 3:**
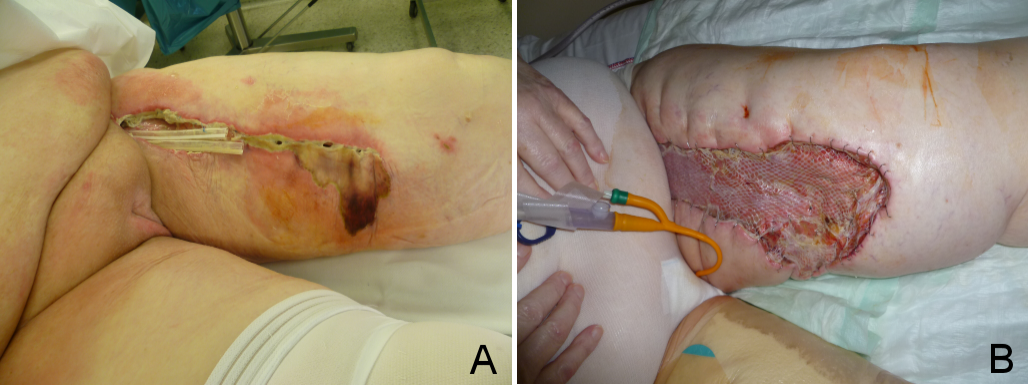
Example of a wound infection/breakdown and necrosis before A) and after B) revision surgery with skin grafting.

Seven/57 patients (12%) in the TissuGlu group, 5/17 (29%) in the ARTISS group, and 12/63 patients (19%) in the control group required wound revision surgery as a result of infected seroma and wound break down development (non-significant) (Figure 3 (Fig. 3)). This represented a 36% reduction in the need for revision surgeries in the TissuGlu group and an increase of 54% in the ARTISS group as compared to the control group. Overall, patients requiring revision surgery underwent a mean of 1.9 procedures to resolve the complication.

### Total drain volume and time to drain removal

There was a lower mean total post-operative drain volume with 444±47 ml (TissuGlu) and 342±59 ml (ARTISS) vs. 524±61 ml (control) (p<0.001 among groups F=83.3, control vs. TissuGlu p<0.001 and comtrol vs. ARTISS, p<0.001).

The mean time to drain removal was 5.9±2.1 days (range 2–11) in the TissuGlu, 4.9±2.4 (range 1–9) in the ARTISS, and 6.5±1.9 (range 2–10) days in the control group (p=0.014 among groups, F=4.4, control vs. TissuGlu p=0.104 and control vs. ARTISS, p<0.005).

**Figure 4:**
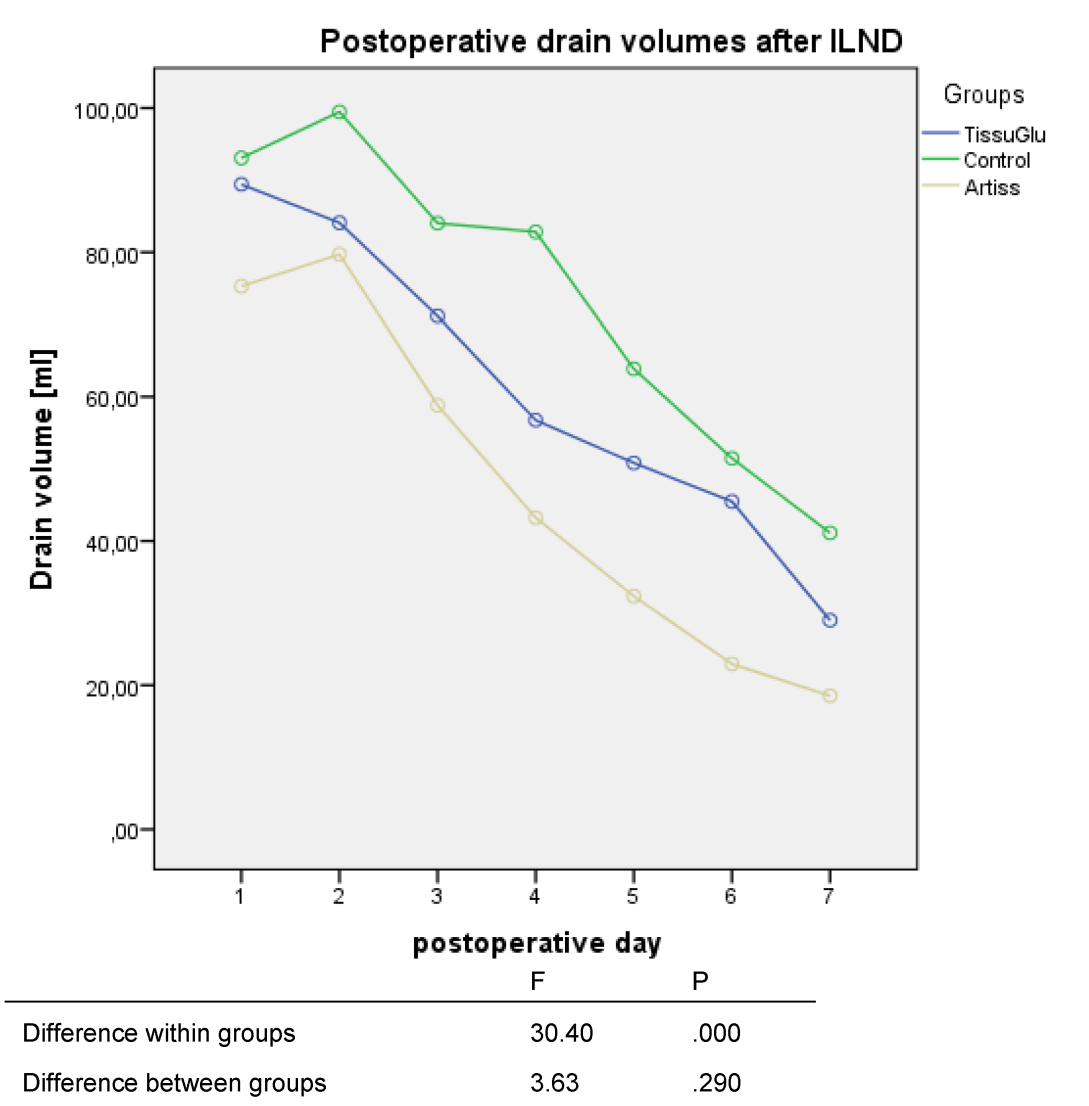
Mean post-operative daily drain volumes after ILND

Post-operative daily drain volumes were highest in the control group followed by the TissuGlu and ARTISS groups as shown in Figure 4 (Fig. 4). In general, the drain volume declined toward post-operative day 7 in all groups. There was a statistically significant lower amount in daily drain volumes for the ARTISS group but not for the TissuGlu group compared to the control group (Figure 4 (Fig. 4)).

Table 2 (Tab. 2) gives a clinical impression of the effect of the surgical adhesives used in the study and shows that the ARTISS group has a high risk reduction concerning extended drainage but a negative effect in terms of risk reduction for infection and the total amount of complications.

To avoid revision surgery once, 15 ILND cases need to be performed with the use of TissuGlu.

The risk for infection and extended drainage is reduced by the use of TissuGlu if 22 and 8 patients respectively are treated.

Within all patients of the study, high BMI was associated with post-operative morbidity. Mean BMI differed significantly when comparing patients with and without complications (Table 3 (Tab. 3)).

## Discussion

The use of TissuGlu adhesive in our ILND patients was associated with a reduction in post-operative wound-related complications. This included reductions in wound infections, the need for extended drainage past day 8, and the need for revision surgery or wound debridement. The initial benefit of lower drain volumes and less extended drainage in the ARTISS group was lost in the higher occurrence of wound infection and revision surgery. As published in other studies, surgical approaches with fibrin sealants have not proven to be successful in the prevention of seroma formation for ILND [12], [13].

The most probable explanation for this phenomenon is the fact that fibrin sealants are completely dissolved by approximately 7 days making rebound seroma formation possible. The adhesive effect of TissuGlu is persistent for 4–6 weeks keeping dead space reduced while wound-healing takes place. Because of the negative results in the pilot study for fibrin sealant in respect to wound infection and revision surgery, we did not further utilize this option. The use of fibrinogen/thrombin-coated collagen sealant patches seems to be a further option in reducing morbidity, yet larger case series should be observed to verify the effect of these products [14], [15]. Another promising alternative seems to be epidermal vacuum assisted closure (VAC) of the groin region as described by Tauber et al. [16]. The authors concluded that VAC might be advantageous for the prevention of post-operative wound complications, but that prospective, controlled studies were necessary to evaluate efficacy and cost-effectiveness.

We can confirm that patients with a high BMI have an increased risk for wound infection as stated by Poos et al. [17]. But high BMI above 29 are also significantly associated with prolonged drainage time and the need for surgical intervention due to wound-associated morbidity as shown by our data. Therefore, before ILND, all patients with a BMI greater than 29 need to be carefully assessed and informed about their additional risk for post-operative morbidity.

This study indicates that the use of the surgical adhesive TissuGlu can reduce post-operative morbidity within a surgical procedure with an extremely high risk for complications. The number needed to treat with this surgical adhesive to avoid the complications infection, extended drainage/seroma and revision surgery is 13. This is a clinically useful measure of the effect of treatment as described by Cook et al. [18]. For comparison, as demonstrated by Herath et al., the NNT with use of continuous prophylactic antibiotics to avoid an exacerbation of chronic obstructive pulmonary disease is 8 [19].

As shown in the data presented by Rimouche et al., morbidity can also be reduced by implementing surgical techniques such as transverse incision, preservation of the long saphenous vein and Sartorius switch. The overall complication rate reported in their own patients in this paper was 61% [20]. This is lower than literature reported complication rates of up to 87%. We found an overall complication rate of 44% in our control group although we perform longitudinal incisions without Sartorius switch and with ligation of the great saphenous vein. We account for the relatively low complication rate due to our strict post-operative management care which is strictly followed within our hospital.

A reduction in post-operative complications requiring surgical intervention is an important factor in terms of reduction of hospital costs but more importantly, by further reducing morbidity after ILND, we hope to influence the outcome and prognosis of Melanoma patients positively by avoiding delays to receiving interferon alpha therapy. Long-term, randomized multicenter outcome studies are necessary to evaluate this hypothesis.

## Conclusion

Our experience demonstrates that the use of TissuGlu was associated with a substantial reduction in complications in comparison to the standard treatment group following ILND. This effect was not demonstrated for the use of the fibrin sealant ARTISS, although we noted a significantly reduced daily drain volume within the first seven days after surgery. Reduction of complications potentially results in improved patient outcomes, a reduction in delays to receiving interferon alpha therapy and a reduction in overall patient care costs related to post-operative physician visits, number of hospital readmissions, and revision surgery procedures. 

We plan to continue using TissuGlu as a standard in ILND and to also utilize this adhesive in other procedures such as latissimus dorsi donor site, which have similar challenges. We will also continue to add patients to this series in order to further assess potential improvements in clinical outcomes associated with this product. 

Limits to the study: The results of post-operative drain volumes are constrained as a result of our clinical practice of removing drains at day 8 and the lack of noting drain volume data beyond this date. 

## Notes

### Competing interests

P.L. Stollwerck and A. Krause-Bergmann received speaker’s honorarium for Cohera^®^ Lunch symposiums at the Congress of the German Plastic Surgery Society (DGPRÄC) in 2012, Bremen, Germany and 2013, Muenster, Germany.

The authors declare that they have no competing interests.

## Figures and Tables

**Table 1:**
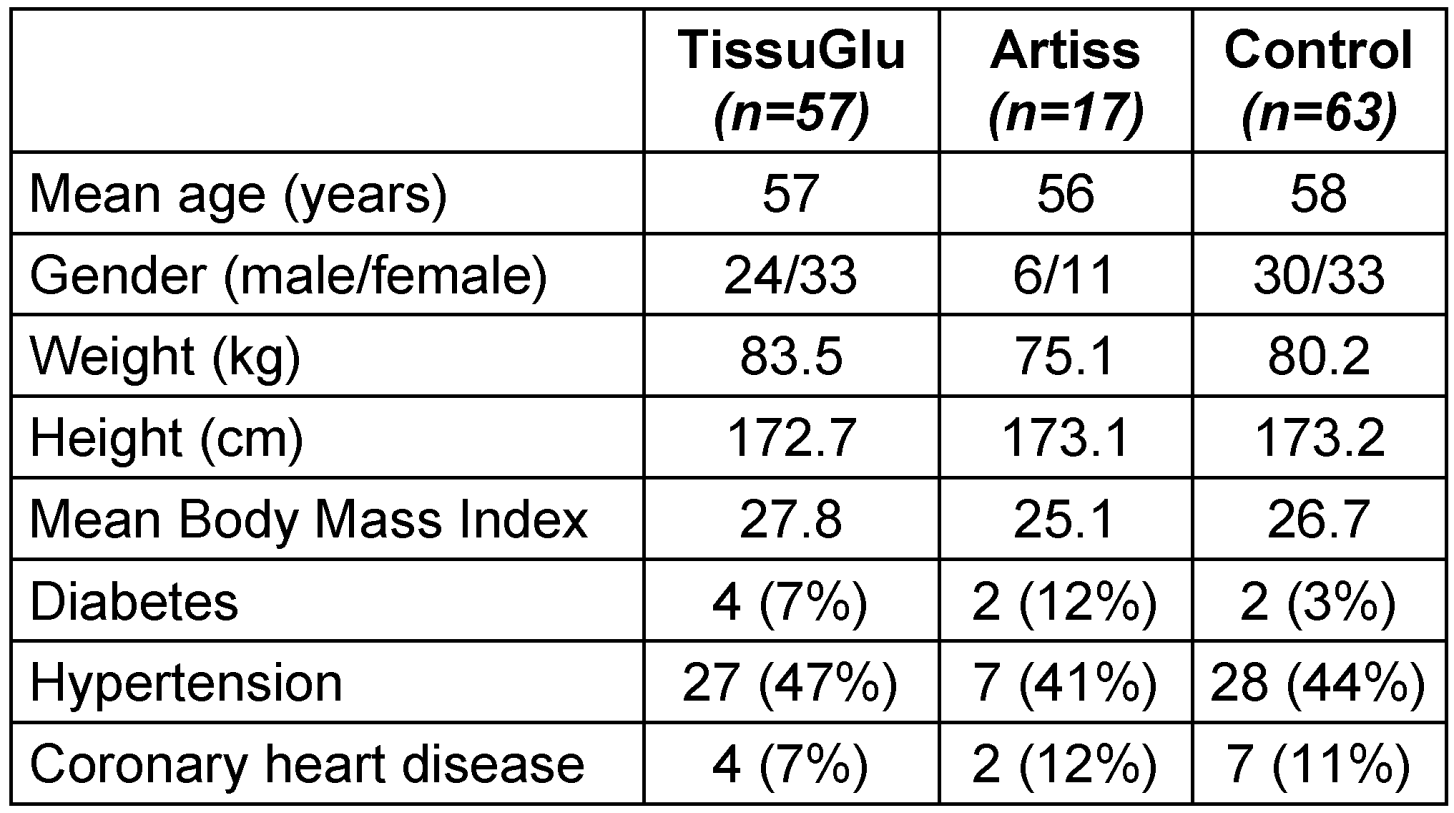
Patient demographics and co-morbidities

**Table 2:**
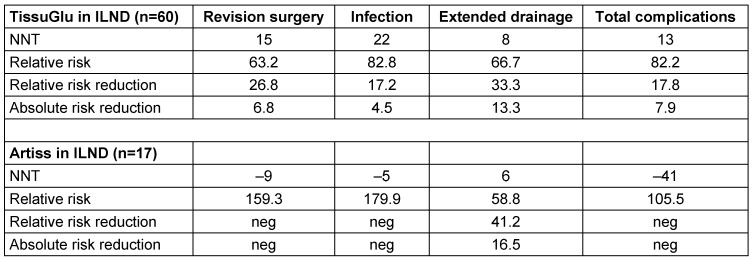
Number needed to treat (NNT) ILND+TissuGlu/ILND+Artiss vs. control

**Table 3:**
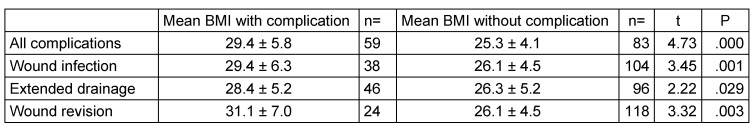
Influence of BMI on post-operative morbidity in ILND (n=137)
